# Mixed adenoneuroendocrine carcinoma of the gastric stump following Billroth II gastrectomy: case report and review of the literature: Adenocarcinoma neuroendócrino misto do coto gástrico após gastrectomia à Billroth II: relato de caso e revisão de literatura

**DOI:** 10.1590/1516-3180.2013.9080911

**Published:** 2015-04-14

**Authors:** Everton Cazzo, Helena Paes de Almeida de Saito

**Affiliations:** I MD, MSc. Assistant Lecturer, Department of Surgery, Faculdade de Ciências Médicas da Universidade Estadual de Campinas (FCM-Unicamp); and Attending Physician, Department of Surgery, Centro Médico de Campinas (CMC), Campinas, São Paulo, Brazil.; II MD. Assistant Lecturer, Department of Internal Medicine, Facudade de Ciências Médicas da Universidade Estadual de Campinas (FCM-Unicamp); and Attending Physician, Department of Clinical Oncology, Centro de Oncologia de Campinas (COC), Campinas, São Paulo, Brazil.

**Keywords:** Stomach neoplasms, Gastric stump, Mixed tumor, malignant, Adenocarcinoma, Neuroendocrine tumors

## Abstract

**CONTEXT:**

Gastric stump cancer after gastric resection is a well-known disease. It may be a newly developed cancer after resection due to benign disease, or recurrent or residual disease after oncological surgery. The predominant histological type is usually adenocarcinoma. This study aimed to report on a rare occurrence of a mixed adenoneuroendocrine carcinoma (MANEC) on the gastric stump.

**CASE REPORT:**

The case of an 83-year-old female who presented a locally aggressive gastric stump MANEC, 35 years after Billroth II gastrectomy to treat a peptic ulcer, is reported. The patient underwent resection and adjuvant therapy. She has been followed up for one year without signs of recurrence.

**CONCLUSION:**

MANEC is a rare type of gastrointestinal neoplasm. The classification, histopathology, clinical features, treatment issues and prognosis are discussed along with a brief review of the literature.

## RESUMO

**CONTEXTO:**

O câncer de coto gástrico após gastrectomia é uma condição extensamente documentada. Pode se tratar de doença desenvolvida após a ressecção por doença benigna, ou ainda doença recorrente ou residual após cirurgia oncológica. Geralmente, o tipo histológico predominante é o adenocarcinoma. Este estudo tem como propósito relatar a rara ocorrência de um adenocarcinoma neuroendócrino misto (MANEC) no coto gástrico.

**RELATO DE CASO:**

É relatado o caso de uma mulher de 83 anos que apresentou um MANEC localmente agressivo 35 anos após uma gastrectomia à Billroth II devido a úlcera péptica. Foi submetida a ressecção e terapia adjuvante e foi seguida por 12 meses sem sinais de recorrência.

**CONCLUSÃO:**

Os MANECs constituem raro tipo de neoplasia gastrointestinal. Sua classificação, histopatologia, aspectos clínicos, tratamento e prognóstico são discutidos junto com uma breve revisão de literatura.

## INTRODUCTION

Occurrences of gastric stump cancer (GSC) after partial gastric resection, especially when Billroth II reconstruction is carried out, are a well-recognized problem.^1^^,^^2^ GSC was classified into three categories by Kidokoro et al.:^3^ cancer newly developed in the remnant stomach; recurrent cancer in the remnant stomach; and cancer remaining in the remnant stomach after the initial gastric surgery. The carcinogenesis of newly developed GSC is strongly associated with chronic duodenogastric reflux of bile and pancreatic juice, and hypochlorhydria secondary to denervation through vagotomy.^1^^,^^3^^,^^4^

Because a great number of younger patients underwent this procedure over the decades prior to the introduction of more effective drug therapies against peptic disease that have been developed lately, a significant number of individuals are exposed to higher risk of gastric cancer nowadays. It is generally reported that chronic degenerative changes to the gastric mucosa lead to development of adenocarcinoma with varying degrees of differentiation.^4^ There are no previously reported cases of mixed adenoneuroendocrine carcinoma (MANEC) arising in the gastric stump subsequent to Billroth II gastrectomy.

## CASE REPORT

An 83-year-old female sought assistance due to weight loss (10 kg), epigastric fullness and regurgitation of solid food over a three-month period. The patient presented mild hypertension that was under control by means of monotherapy (enalapril maleate) and had undergone partial gastric resection due to a peptic ulcer 35 years earlier.

On clinical examination, an epigastric mass was palpated. Rectal and vaginal examinations showed no alterations. From esophagogastroscopy, the following were observed: Billroth II gastrectomy and diffuse thickening and infiltrative appearance of the gastric stump mucosa, from the esophagogastric junction to the gastrojejunostomy site, suggestive of *linitis plastica.* Histopathological examination revealed poorly differentiated adenocarcinoma with signet ring cells. A computed tomography scan showed a thickening of the gastric stump from the cardia to the gastrojejunostomy site; there was apparent invasion of the transverse colon and pancreas tail. Colonoscopy confirmed the presence of transmural invasion of the colon.

The patient underwent open total gastrectomy along with D2 lymphadenectomy, segmental colectomy, distal pancreatectomy, splenectomy, cholecystectomy and Roux-en-Y reconstruction. The operation lasted four hours and the patient was then moved to the intensive care unit. During the postoperative period, the patient presented nosocomial pneumonia that required treated using broad-spectrum antibiotic therapy and developed deep venous thrombosis in her left leg that needed anticoagulation. Enteral nutrition through a jejunostomy was introduced on the fourth day after the operation and oral feeding was started on the tenth day. The patient was discharged from hospital 28 days after surgery, at the time when antibiotic therapy ended and anticoagulation with oral drugs had been achieved.

Histopathological examination on the surgical specimen revealed that it comprised a poorly differentiated, ulcerated adenocarcinoma with signet ring cells, which infiltrated the gastric wall and invaded the colon segment. Angiolymphatic and perineural invasion were present; the surgical margins were free; none of the lymph node presented metastases (0/15); the TNM classification was T4N0 (stage IIIA). Immunohistochemical analysis revealed diffuse positivity for AE1/AE3; 40% of the sample was positive for chromogranin A and synaptophysin, thus characterizing a differentiated neuroendocrine tumor (NET). Hence, the diagnosis of gastric stump intermediate-grade malignant mixed adenoneuroendocrine carcinoma (MANEC) was confirmed, with predominance of the exocrine phenotype.

Adjuvant therapy was used based on the most common tumor cell type, and the most aggressive type (adenocarcinoma) was taken into consideration. We used postoperative bolus-based chemotherapy consisting of a combination of 5-fluorouracil (5-FU) plus leucovorin (LEU), along with sandwiched chemoradiation therapy of a bolus of 5-FU plus LEU as a radiosensitizer.^5^

The patient presented mild symptoms during the first two courses of chemotherapy, such as abdominal pain, nausea and vomiting, but no dose reduction was applied. The third course was administered with concomitant locoregional radiotherapy. After ten days, the patient presented significant gastrointestinal symptoms comprising diarrhea, nausea, vomiting and abdominal pain, and the radiation therapy was suspended. The treatment continued with chemotherapy alone until five cycles had been completed, with mild gastrointestinal symptoms. The patient has been followed up for one year since the surgery, and the last CT scan did not show any signs of recurrence.

## DISCUSSION

MANECs of the gastrointestinal tract are rare tumors.^6^ A wide spectrum of possible combinations of exocrine and neuroendocrine components has been recognized, ranging from adenomas or carcinomas with interspersed neuroendocrine cells to typical NET with a focal exocrine component.^6^^,^^7^ Both the exocrine and the neuroendocrine components may present different morphological characteristics. For the former, these range from adenomas to adenocarcinomas with varying degrees of differentiation, while for the latter, these range from well to poorly differentiated NET.^7^ A tumor is considered to be of mixed exocrine and neuroendocrine type when at least 30% of the lesion is represented by one of the components.^8^

A review of the literature was conducted through an online search for the MeSH terms gastric stump and stomach neoplasms in Medline (via PubMed); and for the MeSH/DeCS terms gastric stump and stomach neoplasms in Lilacs (via Bireme) (Table 1). There were no cases in which the histopathological findings were similar to those reported in this study.

**Table 1.**
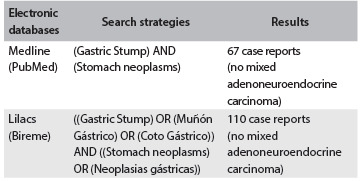
Database search results for mixed adenoneuroendocrine tumor of the gastric stump on July 15, 2014

Mixed exocrine-neuroendocrine neoplasms can be grouped into different prognostic categories according to the grade of malignancy of each component.^6^^,^^7^ They are classified as follows: 1) high-grade malignant MANEC: combination of an adenomatous or carcinomatous component with a poorly differentiated (small, intermediate or large cell type) neuroendocrine carcinoma (NEC); 2) intermediate-grade malignant MANEC: this group includes mixed adenocarcinoma-neuroendocrine tumors (adenocarcinoma/carcinoma that may show different degrees of differentiation, combined with a differentiated NET) and amphicrine carcinoma (a peculiar form of tumor in which the same neoplastic cells coexpress both endocrine and exocrine features, with varying grades of differentiation); 3) low-grade malignant mixed adenoneuroendocrine tumor: neoplasms composed of well-differentiated neuroendocrine and exocrine cells that generally have indolent behavior.^6^

Mixed adenocarcinoma-neuroendocrine tumors, which are in the intermediate-grade category, are formed by areas of tubular, papillary or mucinous adenocarcinoma and areas of grade 1 or 2 NET.^6^^,^^7^ Unlike high grade MANECs, the exocrine component is usually more aggressive than the neuroendocrine component. There are approximately 60 reported cases in the literature, throughout the gastrointestinal tract. They are more prevalent in men in their fifth to sixth decades of life. In the stomach, their distribution is equal in the antrum and in the body.^6^ This group also includes poorly cohesive neuroendocrine carcinoma, in which there are noncohesive signet ring cells mixed with neuroendocrine cells. This subtype often involves the whole stomach with a pattern of *linitis plastica*.^6^^,^^9^^,^^10^

There is no optimal management strategy to date, because of the rarity of this pathological condition.^6^ The most aggressive component should be taken into account when treatment options are considered.^11^^,^^12^^,^^13^ Tumors with a poorly differentiated neuroendocrine carcinoma component must be treated as neuroendocrine carcinomas; tumors composed of an adenocarcinoma along with well-differentiated NET must be treated as adenocarcinoma.^6^^,^^11^ Surgical resection is mostly indicated and must be followed by adjuvant therapy. Early diagnosis is an important factor for avoiding extensive resection like in the case reported in this study.^14^

The prognosis for patients with MANECs is not well-defined.^15^^,^^16^ The data available suggest that high grade MANECs appear to lead to better overall survival than do pure neuroendocrine carcinomas. Intermediate-grade MANECs do not yet have any defined overall prognosis.^6^^,^^17^^,^^18^ Specifically, when the mixed poorly cohesive neuroendocrine carcinoma is presented in *linitis plastica* form, this leads to a poor prognosis, such that most patients die within 10 months of the surgery.^18^ The case reported in the present paper, which was of this subtype, had an unusual outcome, probably due to nonoccurrence of lymph node involvement or distant metastasis, despite the locally aggressive pattern observed. In classical cases of newly developed adenocarcinoma of the gastric stump, the overall survival rate is very similar to what is observed in cases of primary gastric cancer. Conversely, for patients whose prior resection was due to malignant disease, the prognosis is even more somber.^1^^,^^19^

The specific carcinogenetic pathways that lead to mixed exocrine-neuroendocrine lesions are largely unknown.^14^^,^^15^^,^^16^ Whether these can be correlated with the classical mechanisms described for gastric stump cancer development remains to be better understood. The case reported here was a rare malignant neoplasm that developed with a somewhat unusual presentation. Because of the scarcity of data on these specific issues, further study is needed in order to determine issues regarding pathophysiology, treatment options and prognosis, with better accuracy.

## CONCLUSION

Gastric MANECs are rare tumors and this study presents the even more unusual occurrence of MANEC in the gastric stump following Billroth II gastrectomy. Because of the scarcity of such cases, further research is needed in order to determine novel diagnostic and therapeutic approaches.
